# A case of McKusick–Kaufman syndrome with perinatal diagnosis: Case report and literature review

**DOI:** 10.1016/j.amsu.2022.103926

**Published:** 2022-06-06

**Authors:** Irfan Ullah, Shahzad Rauf, Sajjad Ali, Kiran Shafiq Khan, Tayyaba Zahid, Jahanzeb Malik, Rifayat Ullah Afridi, Muhammad Sohaib Asghar

**Affiliations:** a Department of Pediatrics, Kabir Medical College, Gandhara University, Peshawar, Pakistan; b Department of Pediatrics, Khyber Teaching Hospital, Peshawar, Pakistan; c Department of Pediatrics, Dow Medical College, Dow University of Health Sciences, Karachi, Pakistan; d Department of Cardiology, Rawalpindi Institute of Cardiology, Rawalpindi, Pakistan; e Department of Internal Medicine, Dow University of Health Sciences-Ojha Campus, Karachi, Pakistan

**Keywords:** McKusick syndrome, Bardet-beidel syndrome, Hydrometrocolpos, Case report

## Abstract

**Introduction:**

and importance: McKusick–Kaufman syndrome (MKS) is a rarely reported autosomal recessive syndrome characterized by hydrometrocolpos (HMC), polydactyly and various gastrointestinal and renal manifestations.

**Case presentation:**

We present a case of suspected MKS in a prenatal ultrasound with dilated lateral ventricles of the brain and HMC.

**Clinical discussion:**

Main differential diagnosis includes Bardet-Beidel syndrome (BBS) which can present with HMC and polydactyly but retinal manifestations are a differentiating feature from MKS.

**Conclusion:**

Both of the disease syndromes are diagnosed clinically after birth.Keywords: McKusick Syndrome, Bardet-beidel syndrome, hydrometrocolpos, case report.

## Highlights

• McKusick-Kaufman syndrome is a rare autosomal recessive syndrome underreported or underdiagnosed in Pakistan.
• Main differential diagnosis of McKusick-Kaufman syndrome is Bardet-Beidel syndrome. The differentiating feature is ophthalmic lesions and blindness by 20 years.
• Usual diagnosis is made in a postnatal examination with gross abdominal mass, polydactyly, and genital malformation.

## 1 Introduction

McKusick–Kaufman syndrome (MKS) is a rare autosomal recessive syndrome [1]. Hydrometrocolpos (HMC), polydactyly, and congenital heart defects are the hallmark of the syndrome and present in almost 93%–95% of the patients. Hence, also called hydrometrocolpos-polydactyly syndrome [2]. Other manifestations include gastrointestinal and cardiovascular anomalies like ventricular hypertrophy, patent foramen ovale, and the ductus arteriosus. There is lung hypoplasia, renal abnormalities (chronic renal failure and renal cysts), urinary tract abnormalities (hydronephrosis and hydroureter), and ophthalmic lesions [3]. Diagnosis is made clinically [1]. Always suspect MKKS when a female patient presents with HMC and polydactyly or when male patients present with polydactyly with an affected female relative [3]. Fifteen percent of the abdominal masses are caused by a vaginal obstruction in newborn girls [1]. Surgical repair is indicated for obstruction and drainage is done for accumulated fluid [4]. We report a case of McKusick–Kaufman syndrome in a newborn at 6 h after birth with relevant signs and symptoms.

## 2 Case presentation

This female neonate was born at 39 week's gestation to a 35-year-old mother, gravida five and parity five with no previous history of medical interruption of pregnancy. Her prenatal history and laboratory parameters were normal. At 36th week of gestation, and ultrasonography revealed a large abdominal mass as well as dilated lateral ventricles of the brain. She was born through spontaneous vaginal delivery without requiring neonatal resuscitation and an APGAR score of nine at one and 5 min.

**Fig. 1:**
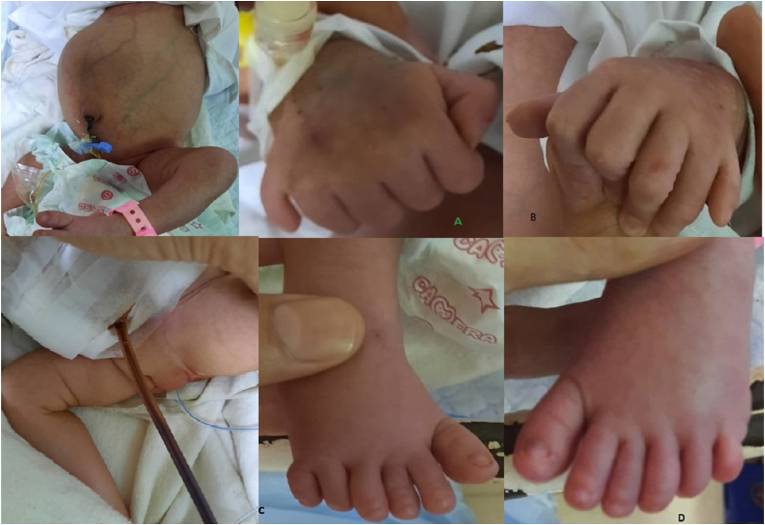
Clinical features of McKusick-Kaufman syndrome with polydactyly of upper and lower extremities, and hydrometrocolpos.

**Fig. 2:**
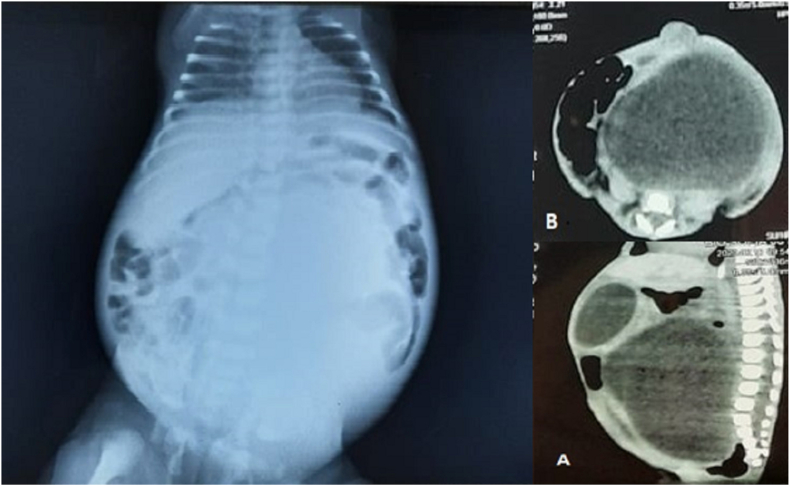
X-ray abdomen and computed tomography showing hydrometrocolpos compressing against the diaphragm causing respiratory distress.

Postnatal examination revealed a birth weight of 2.9 kg, a head circumference of 36 cm, and a length of 43 cm. Facial features were suggestive of trisomy 21. She was in respiratory distress that needed nasal oxygen at 2 L/min. On abdominal examination, there was a large cystic mass with no displacement. Upper and lower extremities had postaxial polydactyly with bilateral pes cavus (Fig. 1). Computed tomography and X-ray abdomen confirmed a pelvic cystic mass between the bladder and rectum, corresponding to a HMC (Fig. 2). Cystoscopy and genitography confirmed HMC and a transverse vaginal septum. Echocardiography showed a patent foramen ovale with non-significant left to right shunt upon crying. On genetic testing there was mutation in MKS gene (604896). No mutation at Bardet-Beidel locus of MKS gene was observed (BBS6; 605231).

**Fig. 3:**
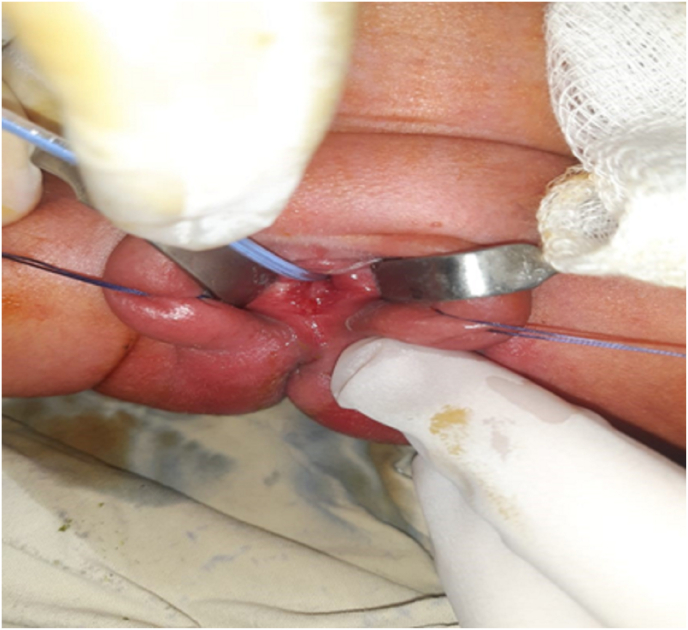
Normal genitalia seen after decompression of uterus.

She was treated with ultrasound-guided aspiration of HMC and approximately 1 L of fluid was drained. Respiratory effort improved after fluid aspiration and a temporary drain was left inside for decompression and she has normal genitalia (Fig. 3). The pediatric surgeon discharged the patient after seven days. Her HMC decreased in size and the patient was doing well on follow-up. Informed consent was taken from the parents for the case report. SCARE guidelines followed in reporting the case [5].

## 3 Discussion

The first case of MKKS in Pakistan was reported by Malik et al., in 2012 [6]. MKS is an underreported perinatal disease in Pakistan because only one case has been reported so far. The first case of MKKS was a 12 years old boy with Tetralogy of Fallot, polydactyly of hands, and hypospadias. There were no ophthalmic findings and differential diagnosis. We should consider a differential diagnosis of MKKS and Bardet-Beidel syndrome (BBS) because HMC and polydactyly are commonly seen in both diseases. BBS is an autosomal recessive condition characterized by retinitis pigmentosa, polydactyly, hypogonadism, learning disabilities, and HMC. Although genital abnormalities may be apparent at birth, other clinical features develop in adolescents. By the age of 20, ophthalmic complications cause blindness [2]. Hence, a newborn with HMC and polydactyly can be diagnosed as MKKS or BBS at birth, but retinal complications can discriminate between the two syndromes early on. In our patient, no retinal anomalies were detected and hence she was diagnosed as MKKS.

MKS is a rare, autosomal recessive disorder that occurs due to a mutation in the MKKS gene at 20p12 locus. HMC is the hallmark of MKKS that presents as a midline large abdominopelvic cystic mass with vaginal atresia or imperforate hymen. Polydactyly or brachydactyly is present in 90% of the cases [1]. HMC obstructs adjacent structures either in prenatal or postnatal life. In a fetus, bladder obstruction may lead to proximal urinary tract dilation that results in oligohydramnios malformation sequence and diaphragm compression, which ultimately results in lung hypoplasia. In utero rectal compression, intestinal obstruction can rarely lead to perforation and peritonitis [7,8]. This usually presents as Hirschsprung's disease [3,9]. Inferior vena cava compression may result in hydrops fetalis and pedal edema [3]. After birth, bladder displacement results in micturition defects which can cause recurrent pyelonephritis and chronic renal failure [8]. Older children or teenagers present with menstrual pain, primary amenorrhea, constipation, lower back pain, or urinary retention [10].

Our patient had no bowel or urinary obstruction and genitalia were normal. However, there was dilation of lateral ventricles of the brain and patent foramen ovale. Some case reports show hydrops fetalis, hydronephrosis, and respiratory failure [[11], [12], [13]]. Once the diagnosis of MKKS is confirmed, immediate decompression of the dilated uterus improves the prognosis for renal and lung capacities [1]. Surgical intervention should be performed early to stop infections in the urinary tract. Molecular genetic analysis may be useful for treating and counseling females with HMC [14].

## 4 Conclusion

In conclusion, these rare syndromes require comprehensive evaluation in the prenatal period by a multidisciplinary team of obstetricians, geneticists, pediatricians, and ophthalmologists. To date, MKKS management involves neonatal assessments only. Its prenatal ultrasonic diagnosis has never been reported. Our case presents prenatal suspicion and postnatal evaluation, management, and treatment of MKKS. We believe our case report of MKKS may contribute to the diagnosis and management in the prenatal period.

## Provenance and peer review

Externally peer reviewed not commissioned.

## Funding

None.

## Consent

Written informed consent was obtained from the parents for publication of this case report and accompanying images. A copy of the written consent is available for review by the Editor-in-Chief of this journal on request.

## Author contribution

S.R, I.U, and K.S.K conceived the idea; T.Z, J.M, R.U.A, and M.S.A collected the data; K.S.K, R.U.A, T.Z, I.U, and I.N did write up of the manuscript; and finally, I.U, M.S.A and S.R reviewed and revised the manuscript for intellectual content critically. All authors approved the final version of the manuscript.

## Ethics statement

All ethical requirements were fulfilled before commencement of study.

## Ethical Approval

Not required.

## Registration of research studies

Name of the registry: Not required.

Unique Identifying number or registration ID: N/A.

Hyperlink to your specific registration (must be publicly accessible and will be checked):

## Guarantor

Muhammad Sohaib Asghar.

## Declaration of competing interest

The authors declare that there is no conflict of Interest.
